# Broadband Power Line Communication for Integration of Energy Sensors within a Smart City Ecosystem

**DOI:** 10.3390/s21103402

**Published:** 2021-05-13

**Authors:** Jan Slacik, Petr Mlynek, Martin Rusz, Petr Musil, Lukas Benesl, Michal Ptacek

**Affiliations:** 1 Department of Telecommunications, Brno University of Technology, Technicka 12, 61600 Brno, Czech Republic; mlynek@vutbr.cz (P.M.); xruszm00@stud.feec.vutbr.cz (M.R.); xmusil56@stud.feec.vutbr.cz (P.M.); xbenes44@stud.feec.vutbr.cz (L.B.); 2 Department of Electrical Power Engineering, Brno University of Technology, Technicka 12, 61600 Brno, Czech Republic; ptacekm@feec.vutbr.cz

**Keywords:** network, broadband, power, line, communication, standard, simulation, adaptive, measurement

## Abstract

The popularity of the Power Line Communication (PLC) system has decreased due to significant deficiencies in the technology itself, even though new wire installation is not required. In particular, regarding the request for high-speed throughput to fulfill smart-grid requirements, Broadband Power Line (BPLC) can be considered. This paper approaches PLC technology as an object of simulation experimentation in the Broadband Power Line Communication (BPLC) area. Several experimental measurements in a real environment are also given. This paper demonstrates these experimental simulation results as potential mechanisms for creating a complex simulation tool for various PLC technologies focusing on communication with end devices such as sensors and meters. The aim is to demonstrate the potential and limits of BPLC technology for implementation in Smart Grids or Smart Metering applications.

## 1. Introduction

Broadband Power Line Communication (BPLC) technology is well known in the commercial and scientific spheres today. It is widespread in areas from home use via smart metering to industry solutions. BPLC technology is a separate part of the generally used term PLC (Power Line Communication) technologies. The main division of PLC technology is given by the frequency band i.e., Narrow Band (NPLC) and Broadband (BPLC) systems.

Specific technology is chosen by the end use application. The real environment affects the functionality of a particular technology. The parameters of the power line (cable type) and the influence of possible interference (background noise) are essential communication parameters. Usability, due to the time and frequency invariance of several parameters such as power spectral density and line impedance, is limited. The predicted suitability of the technology and its specific configuration in particular applications are problematic depending on the specific deployment location. Available technologies resolve this problem by hardcoded settings of adaptive methods in better cases; in worse cases, there is only one configuration that the device works with. As a rule, this is the most robust communication mode. The Signal to Noise Ratio (SNR), or similar related quantities, is considered as one of the main parameters and channel quality indicators. For this reason, the values of this physical quantity are often used as a decision indicator to setup the configuration for communication. In practice this means that when the PLC system recognizes a low value of SNR, it chooses, for example, a modulation mode with fewer states, if this is possible. These processes are not perfect and if the changes are more frequent, the delay or stability of the communication may be even worse than without such an adaptation. Because of this, PLC does not always seem to be the most suitable candidate for a stable connection for high-speed networks. The article does not focus on NPLC, mainly due to limited transmission speed, although NPLC systems are considered in smart grid and smart metering applications [1], presented in articles [2,3,4,5]. NPLC technology is considered in the related works section. The paper is divided into four parts.

The first part deals with approaches and possible ways of developing adaptive methods for PLC technology using a simulator. To create an adaptive method, it is necessary to design topologies in a simulation environment, where it is possible to perform experimental simulations. These simulations are based on mathematical models of the physical parameters of cables, background noise, attenuation and the number of communication devices.

The second part demonstrates available works with experimental simulations of the BPLC technology with focus on smart grid and smart metering areas.

The third part shows comparison of a real measurement and corresponding simulations. The scenarios considered in this work are taken from a real smart grid infrastructure.

The fourth part demonstrates several experimental measurements of PLC systems under real or laboratory conditions. These measurements bring short and long term results in the form of specific data for evaluation of a specific PLC topology as to whether it suits the real infrastructure. Other parts of the paper deal with simulations of real scenarios using the NS-3 simulator. The first results of the simulated scenarios serve to verify the expected trend based on line distance. The second series of results correspond with the real smart grid infrastructure via which the measurements were taken. The main goal of the paper is to present a possible method of low-budget development of PLC systems with verification of suitability for end application before installation.

## 2. State of the Art

The most widespread use of PLC technologies is in smart metering [6,7]. In practice this means, e.g., downloading data from electrometers, communication between distribution transformer stations [8], etc. BPLC technology is today considered, e.g., in automotive application areas [9]. Another case is home use where BPLC is included as a primary or secondary communication channel. In most cases it is the BPLC plug-in adapter which can replace high-speed connection, i.e., Wi-Fi routers in domestic use. The ever-evolving field of smart metering, smart-grid and smart city areas requires an even more advanced system with high precision monitoring, if possible. Restrictions in BPLC technology do not allow such options compared with other technologies. It can be expensive to perform a test implementation of the system without guaranteeing the required functionality because of BPLC’s disadvantages, such as the high channel variability in different conditions. Therefore, it is necessary to develop advanced simulation systems to determine the performance of the communication technology.

### 2.1. Standardization

An important aspect is the standardization of these technologies. The most recognized standards for NPLC are ITU-T G.hnem [10], IEEE 1901.2 [11], PRIME [12,13] and G3-PLC [14,15]. The BPLC systems are supported by fewer standards, e.g., IEEE P1901 [16,17]. The first BPLC standard, approved in 2010, aimed to achieve more than 100 Mbps PHY rate in 100 MHz band for access networks and home area networks.

The most widespread BPLC technology in the commercial sphere are the standards/recommendations of the HomePlug Powerline Alliance [18]. The HomePlug Powerline Alliance has been involved in standardization since 2000. The first series of recommendations HomePlug 1.0 (created according to the first BPL standard—TIA-1113 [19]), HomePlug GreenPHY (mostly used in e-mobility for charging stations), HomePlug AV and HomePlug AV2 are commercially used for HAN as plug-in adapters to extend the internet range.

The latest standard for BPLC technology is ITU-T G.hn [20]. This is made up as a set of specifications G.996x, which are the physical (PHY) layer ITU-T G.9960, link layer ITU-T G.9961, management ITU-T G.9962 (G.hn-mgm), MIMO (Multiple-Input-Multiple-Output) extensions ITU-T G.9963 (G.mimo), PSD management and setting ITU-T G.9964 (G.hn-psd), coexistence with other PLC systems ITU-T G.9972 (G.cx), and remote management ITU-T G.9980 (G.cwmp). These recommendations are described in detail in [21,22]. The baseband for BPLC modems is 2–50 MHz or 2–100 MHz, and the passband is 100–200 MHz. High-frequency bands are also specified for a coaxial cable transmission in the 350–2450 MHz band. As with IEEE 1901.2 and HomePlug, the OFDM (Orthogonal Frequency Division Multiplexing) modulation technique is used. The number of OFDM carriers is 256, 512, 1024, 2048 or 4096. The carrier spacing for PLC is approximately 24,414 kHz. Bigger spacing for the phoneline or coax cables is also defined. Every single carrier can be modulated by 4096-QAM—12 allocated bits. The allowed medium access methods are CSMA/CA (Carrier Sense Multiple Access with Collision Avoidance) and TDMA (Time Division Multiple Access).

Smart grid and smart metering systems require standardized systems where the best option can be considered to be the latest G.hn standard. Today, the P1901 standard is still the most widespread for smart grid and smart metering applications.

### 2.2. Simulation Environment Review

Because there was no specialized tool for construction and simulation of PLC networks in the commercial sphere, the PLC module for the Network Simulator 3 framework (NS-3) [23] was developed at the University of British Columbia in Vancouver. The simulator is primarily intended to design and simulate network technologies.

There are also similar tools in the Matlab environment, but they do not have NS-3 potential and capabilities, especially for the purpose of combining different network technologies in one hybrid topology, i.e., Cañete [24] or FTW [25,26]. The source codes for the FTW simulator are also available for NS-3. The comparison of these simulators is dealt with in [27]. The configuration file (Matlab type) is available for the Cañete simulator only. It is not possible to apply custom model algorithms, but only set values for parameters, which is considerably limiting. All these allow simulation in extensive topologies. These simulators are focused on physical layers (channel frequency response) and RLGC parameters.

The NS-3 framework allows the implementation of library-based communication technologies with the ability to edit communication layers. The PLC framework is open source. The framework allows the definition of different topologies and enables time-variable and frequency-variable behavior of the PLC channel in the simulation. The advantage of NS-3 against the Matlab-based simulator is that there are libraries for many of the communication technologies implemented in NS-3. Therefore, it is possible to combine multiple technologies and simulate, for example, smart grid applications or heterogeneous/hybrid networks.

The PLC module is designed to develop and test applications for power transmission between PLC devices in network topology. The user must specify topology parameters such as geometry, used cable types, impedance values, and location of PLC devices in the network. Ref. [28] shows the importance of modelling a network topology in BPLC. PLC channel response for a typical indoor home network is analyzed.

Refs. [29,30] are based on simulations in the Matlab environment. Ref. [29] is focused on attenuation in low-voltage PLC and creates an OFDM model for comparison of the bit-error rates. In [30], the transfer function, phase response and channel capacity of different indoor power line models are investigated by varying the different parameters of the powerline, focusing on the factors of attenuation and distortion in BPL. Refs. [31,32] demonstrate results using the Network Simulator. Ref. [31] uses the older Network Simulator 2. Ref. [32] demonstrates results of BPL simulation using NS-3 with implemented Standard IEEE P1901 and medium access CSMA/CA. The paper shows in-home simulations where KNX smart appliances are considered, along with the monitored packet losses and network bitrate in a 24-h simulation.

Extensive work in [33] describes in detail the used power line model and performs a number of simulations covering the possibilities of the PLC module for NS-3. This work shows the complex potential of using the PLC module for NS-3. Evidence of this is also seen in [34], which focuses on modelling of the PLC channel in a home PLC environment with cross-layer optimization of PHY and MAC layers, etc. ref. [35,36,37,38,39] are essential for this paper. These papers work with a G.hn standard implementation and other mechanisms to improve communication performance based on the PLC module by G.hn extension in NS-3 (hereinafter, ‘the simulator’). These implementations cover adjustments to PHY, MAC and APP layers. Because the standard is the latest in the BPL area, it is important to improve the simulations.

The overall intent of this article is to demonstrate the simulation results of G.hn communication in order to create a complex simulation tool to predict BPLC communication in a given topology. To achieve the presented results, simulation models of devices and their specific settings based on the G.hn extension were created in [37]. The extension was enhanced by several features to satisfy the requirements of smart metering.

### 2.3. Related Works

A considerable number of papers on the topic of PLC simulations have been published. In this paper, related works are described in five parts. The first part deals with simulations in general. These are mainly comparison studies of network simulators with focus on routing protocols. These works serve as the main motivation for using the NS-3 tool. The current situation of simulation software tools, their usability, usefulness, advantages, and disadvantages are covered. The second and third parts contain works dealing with NPLC and BPLC simulations for smart grid and smart metering systems with the aim of finding relevant simulations of standardized PLC systems. The fourth part contains real measurements of PLC under real conditions with corresponding simulations. This article contributes in this regard with Section 7, where simulations of a real scenario for a smart grid system are provided. Analysis in this part of related works is focused on simulation of standardized systems which correspond with real PLC systems required in the field of smart grid and smart metering. The fifth section on related works analyses several measurements of PLC technology used in a smart grid application. The following list summarizes the points targeted in the analysis:

Section 2.3.1 Simulation in general:

- The need for simulations of communication technologies in general.
- Available simulation tools, their sustainability and potential use.
- The need for further development of simulation tools for PLC technologies.
- Usability of PLC simulations in the field of smart grid and smart metering.

Section 2.3.2 Simulations of BPLC systems:

- Works focusing on simulation models of PLC systems.
- Complex and partial simulations of PLC systems.
- Overview of simulation results in available literature.
- Discussion of the benefits of some works.

Section 2.3.3 Comparison of real measurement and corresponding simulations:

- Simulation in comparison with measurement results of the real topology of a PLC system.
- Simulated infrastructure of smart grid and smart metering architecture.

Section 2.3.4 Measurements of real PLC systems:

- Available measurement results of PLC systems as possible inputs for verification of simulation results.
- Methods for and results of measuring PLC systems under real and laboratory conditions.

Each sub-section above contains one or more tables with clear lists of references according to the key content key of the paper. These tables summarize the analyzed works with reference number, year of publication, brief description of the focus of the work, and the simulation environment. The content type, the targeted type of powerline and the frequency band of the OFDM communication are also included.

#### 2.3.1. Simulation in General

The authors of [40] understand the need for using simulators in the area of communication technologies. They discuss and compare the routing protocols AODV (Ad-hoc On-demand Distance Vector) and DSDV (Destination-Sequenced Distance-Vector Routing) included in simulation tools NS-2 and NS-3. Their focus is on Mobile Ad-hoc NETworks (MANET) [41] and their simulated performance with QoS (Quality of service) parameters such as throughput, delay and Packed Delivery Ratio (PDR). Unlike the PLC technology, MANET uses a wireless medium, but both are influenced by common parameters like signal strength, transmission power, background noise, etc. In both cases the QoS parameters are important, and especially the throughput can be considered as the main parameter examined in simulations.

Paper [42] considers heterogeneous communication channels in designed networks based on PLC and RF. The use of the mesh mode is the most relevant for the design of heterogeneous networks. Similarly to MANET networks, the AODV routing protocol is described in the G3-PLC standard. However, the focus of this article is based on the fact that the AODV protocol was created primarily for wireless networks. Therefore, the G3-PLC standard recommends the LOADng protocol, which is based on the AODV protocol. In order to gain the opportunity to increase demonstrative effectiveness, cut the time used for algorithm debugging, and reduce development costs, the NS-3 environment was used for modelling of heterogeneous networks. The results show that the simulations allowed only superficial assessment of the network and the simulated infrastructure needs to be further refined.

Refs. [40,43] compare features of simulators NS-2 and NS-3, where benefits and limitations are mentioned. In general, the NS-3 tool can be considered the most suitable, especially due to its scope and the possibility of developing extensions, which also provides its disadvantage in terms of implementation complexity. A number of papers have been published on NS-3.

Ref. [44] presents a systematic literature review (SLR) of NS-3 simulator works published from 2009 to 2019. A total of 128 articles and studies are included. Basic analysis of available simulators is carried out. Criteria for the relevance of contributions and the main questions and answers defined. Only 10% of studies have been identified as effective case studies demonstrating the effectiveness of employing a network simulator in real applications. In terms of content type, applicative and conceptual studies are mostly represented. An overall higher number of NS-3 citations has been identified in recent years (since 2015), which suggests an increasing interest in and use of the simulator. Modelling and testing of the wireless mobile ad-hoc network MANET certainly contributes to this fact. Papers dealing with MANET simulations under NS-3 are [45,46,47]. Table 1 shows an overview of papers in which the potential and possibilities of the NS-3 simulator can be observed. Based on published facts on the network simulators, the NS-3 environment can be considered the most suitable of those commercially available for the purpose of simulating PLC systems.

The above analysis shows the suitability of the use of the commercial NS-3 for future development of standardized PLC technologies in terms of communication protocols and building a virtual infrastructure to verify real scenarios of smart grid applications. The first step of every simulation is the implementation of a mathematical model based on the physical parameters of the technology. Ref. [48] explains the need for knowledge of the basic properties of the transmission channel in BPLC technology. The channel transfer function (CTF), levels of background noise and channel capacity are the most significant features to determine the channel model. This topic is also addressed in [49], which focuses mainly on impedance, line length and branches of underground cable PLC systems. Reference [23] describes modelling and calculation of the CTF of the PLC technology in NS-3, where the mathematical definitions of the PLC channel are described. Ref. [50] deals with analysis of the channel transfer function of the PLC module for NS-3. Simulations of several topologies are provided considering BPLC technology. Similar work in [51] provides modelling of BPLC technology based on the PLC HomePlug AV (HPAV) specification, but it is erroneously stated as a standard. The paper describes, inter alia, the communication layers and medium access types of the specification. In the NS-2 environment, an HPAV model was created. This was verified by simulation, resulting in almost the same values as a real HPAV system. On the other hand, a real HPAV system and tested topology were not described as a simulated model.

The aim of the analysis of these articles was to find out how PLC channel analyses are performed, for what purpose, and what is the relevance of the results. It can be stated that these works demonstrate the functionality of the simulator in the given technology by creating a model of the PLC system or describing a real model of the PLC system. When these works deal with the usability of simulators, they always achieve very specific results, especially by setting specific required conditions. Table 2 shows an overview of papers where the topology design considers a specific environment according to the type of communication line.

#### 2.3.2. Simulations of BPLC Systems

This part deals with several published articles on BPLC simulation in the NS-3 environment. Ref. [52] focuses on hybrid communication architectures for distributed smart grid applications. The presented smart grid network contains a combination of five types of communications technology: LoWPAN, PLC, Ethernet, WiFi mesh and WiMAX. The authors created nine NS-3 simulations to verify the performance of all communication technologies in comparison. These comparisons evaluate, for example, BPLC and NPLC network simulations where various models of the channel were used. This work can be considered as a complex design of a smart grid network in NS-3. On the other hand, ref. [48] demonstrates the mathematical relations of a communications channel with focus on overhead medium-voltage topologies with and without lateral branches, which has not been considered in previous work. In [48] the noise on a MV line is described in detail, which plays an important role in the value of the bit-error rate. In this case NS-3 simulations were not provided, but the external electromagnetic (EM) field is examined by focusing on the noise induced. The external EM fields are considered to be the main source of occurring noise on the overhead MV lines and a novel method capable to calculate the corresponding noise levels is presented. The significance of the noise on overhead MV lines is described in paper [53]. Research on the noise characteristics in the frequency range of interest from 40 kHz to 2 MHz is presented.

The authors of [54] aimed to create simulations of the advanced metering infrastructure AMI architecture using BPLC technology and compared this with NPLC simulations. The work considers the real requirements for AMI systems and determines the suitability of BPL and NBPL technology for remote readings from electricity meters. The availability and delay of transmitted data depending on the number of electricity meters in the network are investigated. It also partially presents the issue of security in terms of encrypted communication in comparison with the WPA (AES) standard for WiFi. The goal is to predict the real performance of the system in a particular deployment under the conditions of the real AMI field.

A simple work on PLC simulation in NS-3 can be found in [55], where the description of the developed simulation model is narrow. This article describes the simulations shallowly and summarizes the basic possibilities of PLC simulation in NS-3. An article close to this work is [56], where an advanced G.hn simulation was provided in the frequency band 1.8–30 MHz. The work examines BPLC topology in NS-3 where the channel capacity, depending on the branch length and the impedance, is simulated. The work is also focused on smart metering. In [57], NS-3 simulations in the frequency range 2–30 MHz are presented. The channel capacity was investigated depending on the cable length and the type of modulation technique. Simulated cooperative communication is intended to improve the efficiency and performance of the smart grid network. The simulation itself is created for a real in-home network based on MAC switching.

The last related work [58] describes an adaptive OFDM modulation method for middle-voltage PLC systems in power distribution and utility networks. The paper focuses on fulfilling the delay requirements of the services within the distribution smart grid architecture, grouped according to priority. Distributed energy station control with a delay requirement of under one second is found in the group with high priority. This division into priority groups is defined by the range of the SNR values. The simulation experiments are based on fixed modulation types for each priority group of services and specific OFDM parameter settings where the QoS analysis was provided. The results lead to positive conclusions, where the presented method is recommended in order to improve communication in the band 2–30 MHz of the BPLC systems on the MV lines. Table 3 shows a paper overview of BPLC simulations for smart grid area and channel modelling for specified frequency bands.

#### 2.3.3. Comparison of Real Measurement and Corresponding Simulations

This part deals with papers where the network simulations correlate with a real measurement of a network, such as a smart grid. Ref. [51] was mentioned in Section 2.3.1 as a reference to HPAV simulations in NS-2. The HPAV recommendations are not intended for smart grid application, but the simulations should match real scenarios of BPLC systems. The simulation setup is presented in detail where the medium access method, data segmentation of UDP, TCP traffic and queue processing techniques were used for performance evaluation. The paper presents the developed HPAV model in a validation process by the average throughput comparison of the simulated model and the real system. The real HPAV system configuration is not described and the specific name of the HPAV device is not given, but only the information that the configuration of used devices is based on the default parameters specification of the HPAV. This paper demonstrates the fact that an NS-2 simulator can produce almost the same simulation results as the measured results of a real BPLC system.

Ref. [59] is another paper where the comparison of PLC simulation and real measurement is provided. This examines the standardized NPLC frequency band (CENELEC A) intended for advanced metering infrastructure (AMI). The overhead middle-voltage line was used for testing. Results of the comparison show the change in attenuation depending on the frequency of the simulation and measurement. The maximum deviation between the simulated and measured values is approximately 2 dB. The article does not state a specific method of performing the simulation. The main goal of the work is focused on the problems of measuring and procedures for characterization of MV grids for NPLC.

Ref. [56], described above, is also related to this section. Advanced BPLC simulation of the G.hn standard was performed with topology, which was created on the basis of a real BPLC network. Table 4 shows a paper overview of the works dealing with comparison of performed measurements under real conditions and corresponding simulations.

#### 2.3.4. Measurements of Real PLC Systems

The last part briefly presents several articles in which the measurements of BPLC technology were provided. Ref. [60] describes a hybrid smart grid architecture that consists of a serial bus, ethernet, power line technology (PLC), controller area network (CAN) and wireless communication. Each of these technologies was measured. The NPLC system was installed to an LV underground line, where the transmit amplifier frequency response was monitored in the range 1–20 kHz, which corresponds with the standardized band CENELEC-A.

Experimental measurement presented in [61] focuses on BPLC adapters intended for home use. Used devices meet HomePlug AV2 (HPAV2) recommendations. The goal of the measurement was line throughput comparison of laboratory and office environments on the LV line, where the frequency range 2–86 MHz was used. Standardized RFC methodologies were used for evaluation. Similar work in [8] used IEEE 1901 BPLC modems for measuring throughput in laboratory and real field scenarios. Standardized RFC methodologies were also used for evaluation.

Ref. [62] shows a communication system architecture for the SCADA system where LV smart meters are monitored. NPLC, for which the experimental tests, were provided is partly used in the architecture. These tests have been carried out in the pilot smart grid of the island of Favignana. Other work [63] tested BPLC technologies in the substations of LV and MV lines. The tests were provided under real conditions and the performance requirements were defined according to the IEC 61850 standard [64]. Interesting results show the estimated dependability of MV and LV BPLC systems with values higher than 99.53% for each measured hop. The paper also shows the throughput of LV BPLC modems on the topology using the *iperf* tool [65]. Works that focus on measuring throughput of BPLC systems in the real field are [8,66]. These papers describe the testing methodology in detail and describe the conditions of the measured topology, such as SNR values. Used BPLC devices and measurement setup are also described. The focus of the papers is the suitability of BPLC technology for smart grid applications where remote data acquisition is required. Table 5 shows a paper overview of the works dealing with performed measurements of PLC systems under real conditions.

#### 2.3.5. Conclusion of Related Works

The analysis of four areas of PLC simulations and measurements gives an overview of significant works and creates the main background for this paper. These areas were selected by the content focus on the simulation software of PLC technology, especially BPLC. NPLC simulations are less examined, due to the focus of this paper. Simulation scenarios in the related works are mostly related to smart grid and smart metering infrastructures. In this section, the simulators’ availability, abilities, and features were given. From this, the NS-3 simulator can be considered sufficiently complex and usable for PLC technologies. Table 1 and Table 2 reviews of published works where the potential and application issue of simulators are described.

The next target of the analysis was to find papers where a comparison of simulation of the real topology with the corresponding real measurement is demonstrated. This group of works present a demonstration of real infrastructures with relevant simulations of PLC systems. As they often focus on general verification, an advanced description of the topology or modem setup may be missing. It has also been found that some works vaguely feature a targeted communication medium i.e., low-voltage and medium-voltage. These types of power lines are especially used in smart grids, which is also the reason for the focus of this analysis. The next parameter of the analyzed works was the frequency band for communication. Usually the full range of the band is given without other description or a direct comparison to a possible standard. A very small part of this work considers improvements of the PLC system in terms of adaptation of communication devices adaptation, due to the conditions of the power line.

Therefore, it can be concluded that it is desirable to implement simulations of specific and current PLC systems (which are in demand in the commercial sphere) in order to give rise to work that can contribute to a more accurate assessment of PLC technology/systems before deployment. The last group of analyzed works deal largely with simulations—real measurements of the PLC system. Data from these works should serve as input parameters for the given simulations, provided that specific information on the given topology and location is given. This includes the distances between nodes, the type of cable, the expected temperature, the ambient humidity, and the age of the cables. All these specific parameters affect BPL communication. Therefore, the correct specification of the communication medium at the level of primary and secondary parameters is essential in simulations.

Often, simulation together with on-field measurement on LV voltage is missing or insufficiently published. Accordingly, our own results are also listed. Therefore, this article focuses on throughput simulation for real topology together with on-field measurement for verification of simulation results.

## 3. Motivation and Goals

The previous analysis demonstrates the interest in PLC technologies in both scientific and commercial spheres. The need for simulations is in virtually all technological areas, in order to reduce development and deployment costs. Because there is no universal procedure or tool for PLC technologies to assess the suitability of an implementation, it is necessary to deal with the issue more comprehensively with regard to related parameters that affect the functionality of the technology itself, similar to wireless technologies—signal interference, range, etc. [67] demonstrates a study of unwanted emissions in the CENELEC-A band generated by distributed energy resources and their influence over the NPLC system. Similar work [68] deals with measurement techniques for non-intentional emission above 2 kHz, in order to contribute to the standardization of emission requirements in the frequency band of CENELEC or FCC for NPLC systems.

This work focuses mainly on BPLC technology over MV power lines in the fields of smart grid and smart metering. The MV power line is considered for distribution transformer substation automatization, and the BPLC is used for control and data acquisition from sensors and meters in substations. In comparison with low voltage lines, the MV lines show less attenuation on the line, invariant behavior in time, simpler topology without branches on the main transmission line, and low number of sources of interference [54].

The main goal of this work is a demonstration of the BPLC simulator compliant with the G.hn standard. Various simulation methods were performed with respect to transmission efficiency. These methods are described, and the results of simulations are demonstrated. The created simulation scenarios correspond with infrastructures under laboratory and real conditions. The simulation under real conditions is supplemented by specific measurements for comparison. The results also demonstrate a simple comparison of simulation and measurement on one real topology. The aim was to simulate the same trend of transmission speed as was observed in the real environment. For this purpose, the simulations were performed mainly on the application layer.

BPLC is considered for connection of sensors, power monitors and meters for automatization and control of distribution substations. Especially for underground substations, BPLC is the only communication solution after the optical network infrastructure has been built.

## 4. Theoretical Background and Simulation Methodology

**Figure 1:**
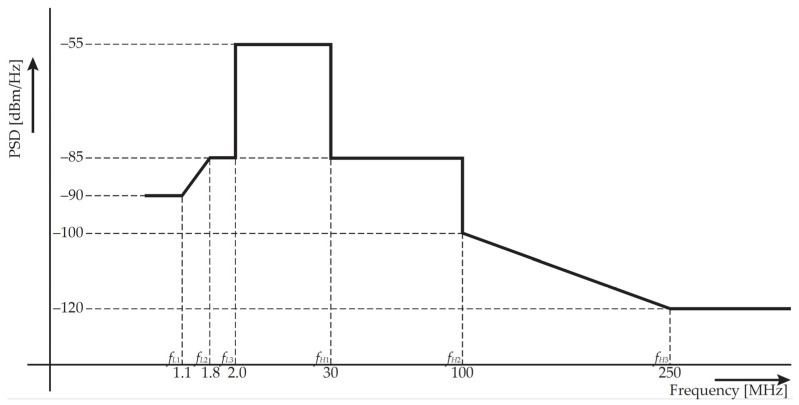
Transmission power regulation according to specification G.9964.

The power spectral density (PSD) according to G.9964 recommendation [69] is implemented in the simulator. Figure 1 shows the PSD according to the relevant frequency band for BPL systems. The implementation allows variable setup of PSD in *f_L1_-f_L3_* and *f_H1_-f_H3_* spectrum fields. Maximum values are restricted by the recommendation.

### 4.1. Choice of Approach to Channel Capacity Calculation

The NS-3 simulator allows two methods of channel capacity calculation. Methodologies of both algorithms were compared and described by demonstration results in order to choose the method for the simulations.

#### 4.1.1. Dynamic Bit Allocation by Bit-Error Rate

This is a method of adaptive bit-loading used in multi-carrier transmission systems, where it is necessary to assign a different type of modulation to each subcarrier. This method makes it possible to reduce the BER for individual subcarriers based on the signal-to-noise ratio by using a lower degree of modulation for modulating the subcarrier at a lower SNR value. (i.e., from 256-QAM to 64-QAM) [70,71]. Higher throughput is possible with a higher number of modulation states, but with a higher probability of bit and symbol errors. The theoretical bit-error rate *P_b_* is defined by the SNR and *M* states of the modulation technique. It is possible to calculate the AWGN channel by the equation [72,73]:

(1) $$BER\approx P_{b}=\frac{\sqrt{M}-1}{\sqrt{M}\log_{2}\sqrt{M}}erfc\left(\sqrt{\frac{3\log_{2}M}{2\left(M-1\right)}\times \frac{S}{N}}\right)\left[-\right],$$

where erfc is an additional error function related to the Gaussian error function erf (Nandagopal, 2011) [74]:

(2) $$erfc\left(x\right)=1-erf\left(x\right)=\frac{2}{\sqrt{\pi }}\int_{x}^{\infty }e^{-t^{2}}dt.$$

Theoretical verification of the QAM-*M* modulations with target bit-error rates is shown in Table 6. The SNR values with defined targets of the $P_{b}$ were simulated in NS-3.

The dynamic bit allocation method uses the algorithm based on a defined limit value of BER (*P_T_*). The algorithm examines reaching the limit of this error rate using a specific QAM modulation. The following equation is used to assess the bit error rate:

(3) $$P=\frac{\sum_{k=0}^{N-1}q\left[k\right]c\left[k\right]}{\sum_{k=0}^{N-1}q\left[k\right]}\leq P_{T},$$

where *k* defines the examined subcarrier; *q*[*k*] is the possible number of bits for the sub-carrier based on the modulation type (i.e., 256-QAM allows transfer of 8 bits); and *c*[*k*] defines the bit-error rate of the current subcarrier calculated according to the equation:

(4) $$c\left[k\right]=\frac{\left(q\left[k\right]\right)-C}{q\left[k\right]-1},$$

where *C* is the capacity of one sub-channel by the Shannon-Hartley theorem [75]. The calculation algorithm for the bit allocation table is described in [70] as follows:

1. Initialization—modulation type assigning for all available sub-carriers.
2. Determine the number of modulation bits [*k*] and BER [*k*] values for all available subcarriers.
3. Calculation and comparison of *P* and *P_T_* by Equation (3). If *P* is less then *P_T_*, the current allocation is left, and the algorithm is done. Otherwise, it continues.
4. Find the *k*-th subcarrier with the highest BER value and reduce the modulation of *k* by one level which decreases the total value of *P*.
5. Recalculation of [*k*] and [*k*] for all subcarriers where the allocation was changed, and then loop from the third step until *P* ≤ *P_T_*.

To ensure a lower value of the bit-error rate, the simulator necessarily reduces the modulation of the subcarriers. Mapping data of bit-error rates and setting of BER values and coding schemes are used for achieving actual values of the bit-error rate BER.

#### 4.1.2. Dynamic Spectrum Management by the Water-Filling Algorithm

**Figure 2:**
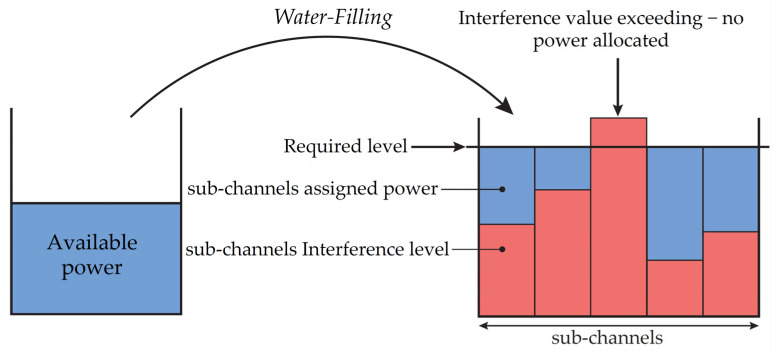
Principle of Water-Filling algorithm [76].

The Water-Filling method is based on adjusting the spectrum of the PSD transmission mask to achieve the most efficient use of the given frequency band. The principle consists in allocating a higher level of transmission power (its distribution) to subchannels with a high SNR value. Conversely, subchannels with a low signal-to-noise ratio can be eliminated by this system [76]. The power allocation is illustrated in Figure 2.

The NS-3 simulator uses the Water-Filling algorithm as follows:

1. Calculation of the required level for all available sub-channels.
2. Iterations through the performed areas triggered by changed PSD values.
3. For all subchannels belonging to one area characterized by the same value of transmission power, the total available power is determined.
4. Allocation of available power for all subchannels to the required level.
5. Calculation of the SNR value according to the updated allocated PSD.

**Figure 3:**
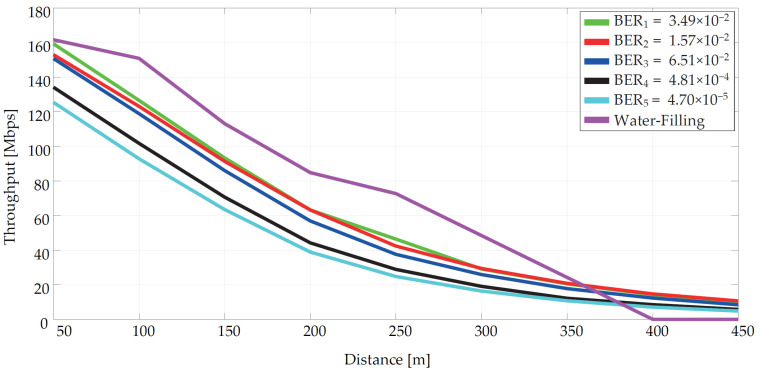
Dependence of transmission capacity on distance for different BER values with the method of dynamic bit allocation by bite-error rate and dynamic spectrum management by the Water-Filling algorithm (purple line).

By obtaining the SNR value, it is possible to calculate the transmission capacity of the channel using the Shannon-Hartley theorem. Figure 3 shows a comparison of the dynamic bit allocation by BER and the dynamic spectrum management by the Water-Filling algorithm.

According to the results, higher transmission capacity can be achieved by using the dynamic spectrum management method, especially in short-distance areas, which is close to real conditions. By the method of dynamic bit allocation by bit-error rate, it is possible to achieve the highest possible communication distance at the required BER value. This important for smart metering systems, which is why in the following text only dynamic bit allocation by BER will be assumed.

The simulator allows simulation for the 25, 50 and 100 MHz frequency bands. For these values, the maximum level of the PSD transmitter according to ITU-T G.9964 [77] and OFDM parameters according to ITU-T G.9960 [22] are defined in the simulator.

The throughput difference according to the communication band was also simulated. Achieved values served to demonstrate the correct trend with respect to the distance in the point-to-point topology. Comparison of both methods is presented in Figure 3.

**Figure 4:**
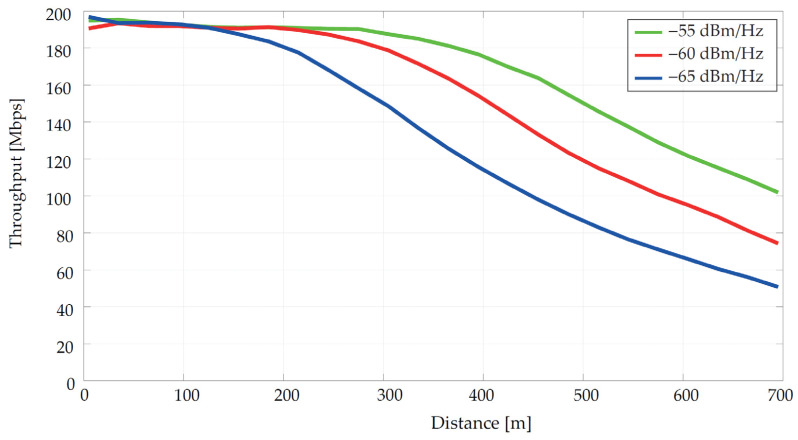
Dependence of transmission speed on distance at different power values.

The communication speed (throughput) is shown in Figure 4 for a point-to-point communication where the PSD was decreased from the maximum allowed value for the 2–30 MHz frequency spectrum. For the highest set value of power −55 dBm/Hz, the transmission speed began to decrease at a distance of approximately 300 m; for lower values this decrease occurs earlier. It is important to note that in a real BPLC topology without repeaters, 300 m can be considered as the limit for stable communication [78,79]. An analogy to reducing performance may be to increase the background noise level. In both cases, the SINR value changes, which in the simulator represents the signal to interference and noise ratio. These values are then used to calculate the capacity/throughput of the line.

There are two methods to choose from for calculating the capacity. The first method is the dynamic bit allocation by bit-error rate (BER) and the second is the dynamic spectrum management by the Water-Filling algorithm.

## 5. Experimental Simulation Scenarios Topology

Two types of simulation were performed. The first part of the simulation considers LV lines, where the throughput is monitored for the verification of the expected trend due to the line distance (Section 6.1, Section 6.2 and Section 6.3). The second part considers MV lines with an experimental simulation setup using a G.hn implementation, where the input parameters are comparable with the IEEE P1901 compliant BPL modems (Section 7).

### 5.1. Low-Voltage Power Line

The first series of presented simulations follows selected parameters of the BPL communication due to the distance between two virtual BPL modems. The results show several experimental G.hn BPL communications with parametric sweep. The simulation targets were to verify the effects of the parameters on the throughput, and how they depend on the low-voltage line distance. For simplicity, only the results of the communication between the two nodes are demonstrated. The presented results are divided by variable parameters:

- Modulation sweep—defined QAM modulations with decision points from 4 to 4096.
- Transmit power spectral density (PSD) sweep—based on G.hn definition for the NS-3 spectral model in dBm/Hz.
- Cable cross-section sweep—cable type NAYY for fixed underground installation. NAYY AxB SE where A is the number of cores and B is the nominal cross-section [mm].
- Cable type sweep—cable types designed for distribution in buildings and underground installation.
- Bandwidth sweep—used bandwidths 25, 50 and 100 MHz correspond to the G.hn standard.

**Figure 5:**
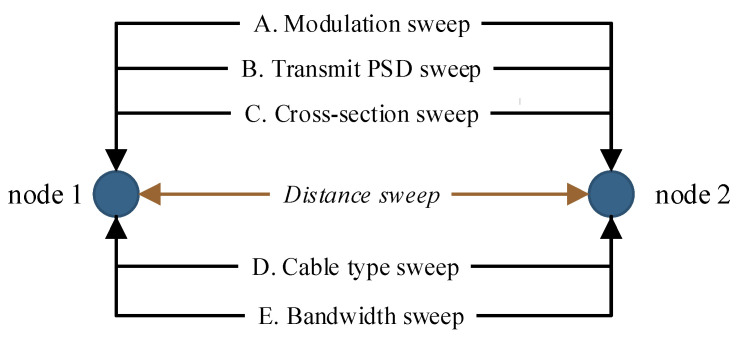
Simulation setup—swept parameters in point-to-point topology.

Each simulation case uses a G.hn extension that has been modified for simulation purposes. The medium access method is common—CSMA/CA. The point-to-point communication system is full-duplex. The statistical method delta is used to determine the application rate with a confidence interval of 95%. The distance sweep at a step of 30 m. The impedance of both nodes is 100 Ω. Summary of the simulation setup is seen in Figure 5.

### 5.2. Medium-Voltage Power Line

**Figure 6:**
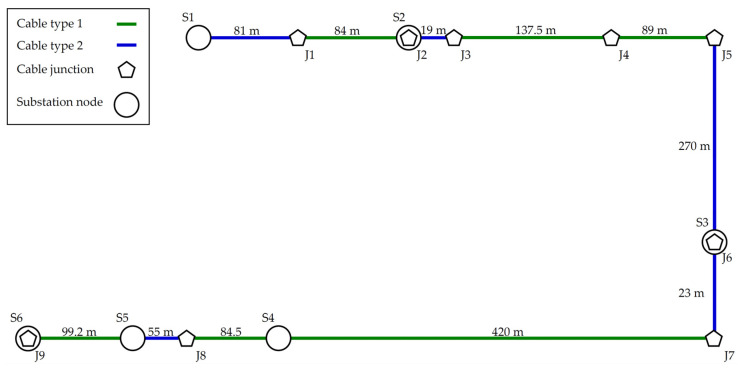
Diagram of real MV power lines with BPLC communication.

This scenario is based on the real topology of a smart grid application. The topology consists of underground power cables for the energy distribution and distribution substations. Figure 6 show this topology with all substation nodes (S1–S6) which are connected by the cable junctions (J1–J9). These junctions create crossroads for other power line connections of the distribution network. From the network structure point of view, two types of cables are used in this case. Cable type 1 is AXEKCY, and cable type 2 is AXEKVCEY. Both types of power line are designed for middle-voltage power transmission.

The NS-3 simulator does not define similar types of MV cables, so they were created for this simulation. Table 7 shows selected values of the cable primary parameters. The values were approximated from available sources and from the catalogue of the cable manufacturer.

As stated in papers [80,81], the frequency-dependent primary parameter is the resistance R′.

(5) $$R\prime \left(f\right)=A_{R}f^{2}+B_{R}f+C_{R}\;\left[\frac{\Omega }{m}\right],$$

where $A_{R}$, $B_{R}$ and $C_{R}$ are the coefficients of the polynomial function and *f* is the frequency. Inductance *L*′ and capacitance *C*′ are frequency independent:

(6) $$L\prime \left(f\right)=L\;\left[\frac{\Omega }{m}\right],$$

(7) $$C\prime \left(f\right)=C\;\left[\frac{F}{m}\right].$$

The parameter conductance *G*′ is neglected in the simulations and only resistance is considered. The only difference between the cables in Table 7 is the electrical isolation which has an effect on the noise interference.

## 6. Experimental Simulation Results

This section describes the achieved experimental results of the simulations. The first part of the simulations considers LV lines, where the throughput trend is monitored, based on the change in PSD, cable type, and bandwidth. Therefore, the target values of throughput were not set for these simulations, but only the verification of the expected trend was considered. These results made it possible to design a simulation of a real topology and real BPL devices, which were experimentally verified in practice by measurements. The second part of the simulations considers MV lines with experimental simulation setup using G.hn implementation, where the input parameters were taken from the IEEE P1901 compliant BPL modems.

### 6.1. Modulation Sweep and Physical and Application Throughput Simulation

The first simulation considers all available modulations. Figure 2 demonstrates BPL throughput on the application layer. The communication includes the UDP/IP protocol, thus the shown rate is considered as the application rate. Input parameters for simulation are described in Table 8.

**Figure 7:**
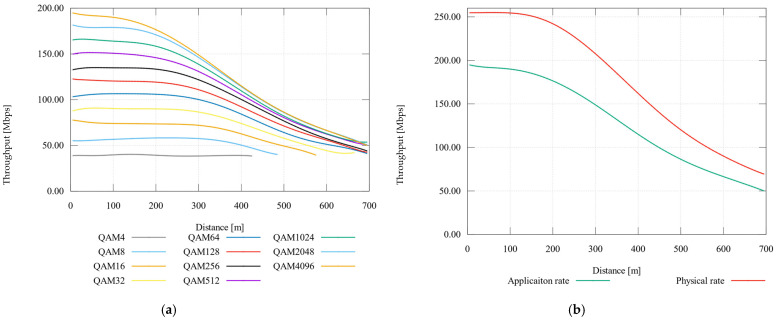
(**a**) Comparison of throughput with modulation sweep; (**b**) Comparison of application and physical rate—QAM4096.

Figure 7a displays the results of the modulation sweep simulation. The QAM4 modulation has tan application rate around 40 Mbps with a reach of approximately 40 m. With multi-state modulation QAM8 the range increased by 60 m and with QAM16 by 150 m from QAM4. A significant throughput drop was reached at 200 m, when the difference of the two closest modulations was approximately 20 Mbps.

Figure 7b displays the comparison of physical and application rate with QAM4096 modulation. The UDP frame headers show the differences of values, where the biggest was 54 Mbps.

### 6.2. Transmitted Power Spectral Density Sweep and Cable Cross-Section Sweep

**Figure 8:**
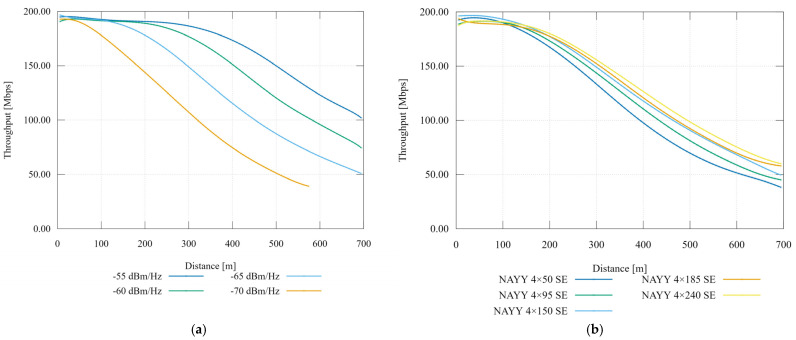
(**a**) Comparison of throughput with transmit PSD sweep; (**b**) Comparison of throughput with cable cross-section sweep.

Recommendation ITU-T G.9964 [20] deals with PSD. In the power line case, in the 25 MHz band the transmit PSD is limited to −55 dBm/Hz. This value has been selected as a reference and is gradually reduced. Table 9 describes the input parameters including used cable types. The NAYY cables are designed for underground installations. Figure 8 displays the results’ maximal transmitting PSD values.

For the highest value −55 dBm/Hz, the application rate has begun to decrease at approximately 300 m according to Figure 8a. With the reduction of transmitting power, the range of communication decreases, where the lowest value was simulated at 600 m. The parallel of transmit PSD reduction can be an increase of the background noise power which is constant in time and frequency domain.

Figure 8b displays the results of the cross-section sweep simulation. Due to a smaller cross-section, the ranges of communication and the application rate are reduced. Because of the change in the cross section, the primary and secondary parameters of the line change. The rate difference of NAYY 4 × 50 SE and NAYY 4 × 240 SE at 500 m is 28 Mbps.

### 6.3. Cable Type Sweep and Bandwidth Sweep

For calculation of the primary and secondary parameters of the CYKY cable, the geometrical properties were used, i.e., the radius of the conductor and the distance between the centres of the cores available from the manufacturer. For the simulation, a lower background noise value was selected at −100 dBm/Hz. In addition, the throughput is monitored only at a distance of 300 m. The rest of the input parameters coincide with the previous simulation as described in Table 10. The simulation output is once again the dependence of the band rate on the distance in Table 10. The next simulation is focused on the influence of the bandwidth on the application rate. The G.hn PLC module defines 25, 50 a 100 MHz bands. Table 10 also shows the PSD values defined for these bands. The maximum transmit power is defined in the simulator by ITU-T G.9964 and the OFDM parameters satisfy the ITU-T G.9960 recommendation.

**Figure 9:**
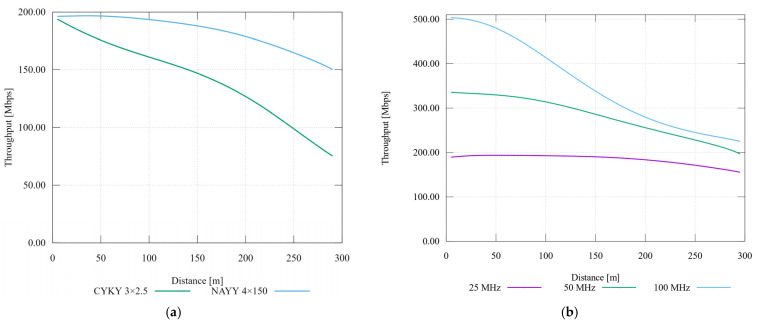
(**a**) Comparison of throughput with cable type sweep; (**b**) Comparison of throughput with bandwidth sweep.

Throughput differences of the LV and MV cables is shown in Figure 9a.The point-to-point transmission rate for different frequency bands is shown in Figure 9b. According to the figure, the largest bandwidth reaches a transmission speed up to 500 Mbps, and for the 50 MHz band the speed is up to 330 Mbps. However, with increasing distance, the speed decreases rapidly, and at a distance of 200 m, the difference is less than 80 Mbps and gradually approaches the lowest bandwidth. This decrease is mainly due to the strong frequency dependence of the primary parameters on the transmission line. The results were reached by the experimental setup for the trend verification of the throughput. Because of this, it is difficult to compare these results with real equipment, especially due to inaccuracies in the consideration of attenuation on the simulated route.

### 6.4. Evaluation of Simulation Results

The above results approach the PLC simulations issue in the BPL area for low-voltage networks. The purpose of this paper is to demonstrate the potential of the NS-3 framework in cooperation with the PLC module and the G.hn extension. Presented results serve towards the development of complex PLC simulators with advanced possibilities of PLC network models including PLC devices, parameterized power lines and overall topology.

The first use case of this simulator is oriented to making predictions based on statistical data, which will be used to measure real topologies, to some extent without the need of a real measurement. The main area of application is in smart-grid networks and smart-metering. The second use case is the design and testing of sub-sections of smart-cities, smart-home or industry 4.0. The third use case is for developing adaptive methods of PLC communication generally, intended for broadband power line technologies (ITU-T G.hn, IEEE P1901, HomePlug AV2) and narrowband power line technologies (PRIME, G3-PLC, ITU-T G.hnem).

The achieved results demonstrate their usability with measurements under real conditions. This is ensured by verifying the theoretical assumptions of basic simulations mentioned in this paper and focusing on the influence of several basic parameters on the application rate in UDP/IP networks. Future work will be focused on improving the simulators for advanced topologies in accordance with PLC under real conditions, especially in smart-grid areas for power distribution and management. Different calculation and definition methods were created for the G.hn PLC module in NS-3. The next steps in this work should improve the overall behavior of the simulated PLC network. This creates a possibility of implementing predictive algorithms, for a specifically defined environment. The main goal of the results was achieving the correct trend of simulations, corresponding to theoretical assumptions which allows the simulation of real smart grid systems under real conditions in comparison with measurement.

## 7. Simulation of Real Scenario and Comparison with Real Measurement

The series of measurements were performed under real conditions on the medium-voltage (MV) powerline. The measurements were performed between all substation nodes in the trace. The aim was to measure the point-to-point communication without repeaters and to find the effect of distance on transmission parameters. The target parameter was the throughput at the application layer.

**Figure 10:**
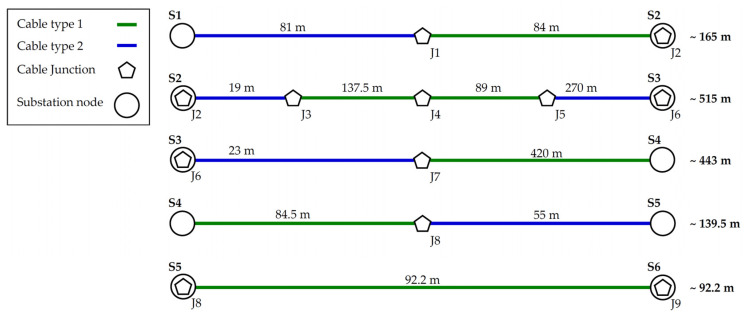
Diagram of the measured and simulated topology with full distance between substations.

The MV cable routes are composed of several types of cables of different lengths (see Figure 10). In the case of the S2–S3 route, the route contains three junctions with four cables. The topological conditions between the substations are defined according to Figure 6. The maximum number of junctions on one trace was five, where two were a part of the substation (immediate nearness of the substation switchboard). The longest measured trace of this topology was approximately 515 m. At the time of measurement, it was not possible to determine more precisely conditions such as the temperature and ambient humidity of individual junctions or cables. According to our own observations from the long-term measurements over a number of weeks, we found a change in the transmission rate, loss of transmission and other parameters depending on the day and time of communication (humidity after rain could be the reason).

EXFO FTB-Pro instruments which were connected to BPL modems via the Ethernet interface were always used for measurements. The measurement methodologies RFC2544 were employed. The achieved values of throughput (transmission speed on a UDP communication layer) are always the average values for the entire measurement period.

The RFC2544 methodology measures the parameters on the third layer of the ISO/OSI model—the network layer. The methodology is based on the UDP protocol, i.e., an unreliable protocol. The testing methodology allows the creation of multiple data streams with different specifications within the Ethernet interface. In the case of these results, the configuration was always optimized for the optimal value of maximum throughput, to achieve the minimum error rate. Furthermore, it is necessary to define the size of the transmitted frame for each data stream. In total, the measurement was performed when transmitting one stream with a frame size of 256 bytes and when transmitting three streams with a size of 256, 512 and 1024 bytes. Two series of measurements using the RFC2544 methodology were performed to test the throughput of the simulated UDP traffic in one direction and to test the response of communication by looping nodes.

**Figure 11:**
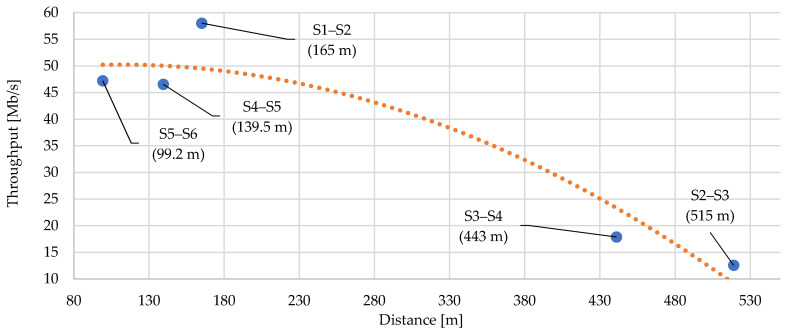
Impact of distance on UDP/IP throughput with the theoretical trend line interpolation.

**Figure 12:**
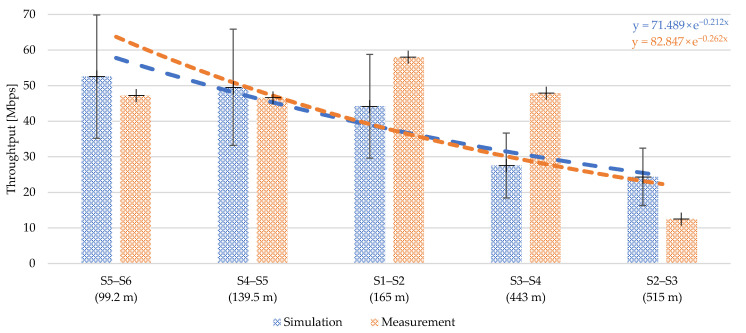
Comparison of simulated and measured results: (Simulation) NS-3 simulation of real topology with PLC model setup due to BPLC modems parameters; (Measurement) Throughput measurement on UDP layer in one direction in sub-sections—without repeaters.

Figure 11 shows the throughputs of UDP-based communication in one direction in sub-sections (point-to-point communication). Figure 12 shows the impact of distance on throughput. The order is not geographically correct, as the topology in Figure 10 shows, but the sections are sorted according to their distances from the shortest to the longest. The important point is that traces do not consist of single cables, but of several different cables with separate junctions. It is possible to see the impact of real variable conditions.

Deviation of measured values of throughput from the theoretical trend line interpolation can be observed. The theoretical trend line is applicable for expected results of the BPLC technology on MV lines. The detected differences may be caused mainly by the variability of the environment and the specific parameters of all elements in the communication path, such as the specified ambient humidity and the age of cables and junctions. Based on this measurements and knowledge of physical cable parameters (cable age, junction, cable type), the precondition for BPLC installation in another area is known. The achieved speeds are considered sufficient within the deployed smart grid solution in terms of the throughput and the reliability of communication in the infrastructure.

### Simulation Comparison with Real Measurement

Based on the measurement results, a simulation was performed, which was configured according to real parameters to provide comparison of simulation with measurements. If comparison shows compliance, the simulation could be used for different allocation or scenarios. First, the topology parameters such as substations distance, sub-section routes and the primary parameters of the cables were implemented. Second, the parameters of the BPLC modems were defined in a simulation model within the G.hn standard (real modems were IEEE P1901 compliant). The weakness of the simulation is the inaccurate definition of the physical layer, whose parameters could not be determined from the used modems. The physical layer setup of the simulation was the experimental setup used in LV simulations (Section 6.1, Section 6.2 and Section 6.3), excluding cable definitions. There are also differences in the setup of the OFDM technique that could not be determined. Table 11 shows a summary of channel parameters for the simulation. Impedance matching was also considered as an input parameter. The load impedance was set to 100 Ω and the input impedance to 40 Ω. These values are also theoretical from the IEEE P1901 standard. Branches were not considered.

The simulation results of the throughput at the application layer are demonstrated together with the measurement results in Figure 12. The results correspond with the defined topology with separate sections—the simulated value always corresponds to a separate route between two neighboring nodes/substations. The exponential expression of interpolation of values is shown for the simulated and measured values.

In the comparison of simulated and measured throughput values there are differences of approximately 26%. The simulation bars in Figure 12 show an approximate error deviation value of 30%. From the achieved simulation results, throughput values at the physical layer were calculated.

**Figure 13:**
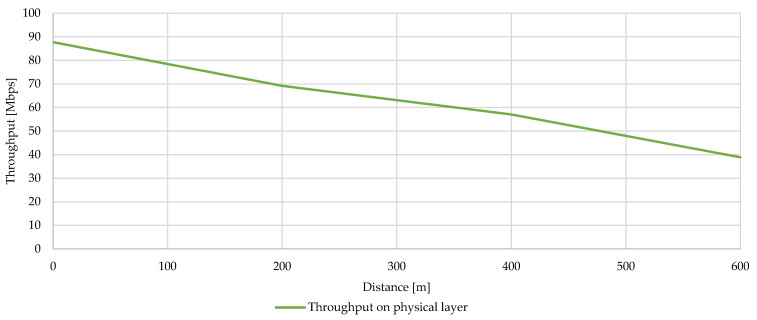
Calculated throughput at the physical layer.

Calculation of the physical rate is based on the throughput values reached, always from one way communication from a more distant node/substation, i.e., S2 to S1 etc. Figure 13 shows the calculated throughput at the physical layer.

## 8. Lesson Learned

For future implementation of BPLC to the NS-3 simulation framework, these recommendations should be used:

- Maximum distance without repeaters.
- Simulation could be the first quick and effective tool for measurement of throughput etc.
- G.hn provides best variant of a standardized solution for BPLC technology.
- Results must be in correlation with physical parameters of cable (age of cables, ambient humidity, temperature, parameters of junctions).

## 9. Conclusions

The performed analysis in four parts of PLC simulations (mentioned in Section 2.3) and measurements provides an overview of significant works and creates the main background for this paper. These areas were selected by the content focus on simulation software of PLC technology, especially the BPLC and NPLC simulations with a minority share. Simulation scenarios in the related works are mostly related to smart grid or smart metering infrastructures. One of the main conclusions of the analysis was the current need for simulations of PLC technologies for specific infrastructures, for example in electricity distribution networks via LV or MV.

The aim of the analysis was to examine the used simulation environments and types of simulations (type of medium, frequency range). One of the main conclusions of the analysis was that in the last 10 or more years there is only one popular and sufficiently comprehensive tool for simulating PLC technologies—NS-3. Works that dealt with simulations marginally and in more depth were presented. The individual outputs of the papers were found to be of high potential use as a tool for effective simulation of PLC technologies. However, the problem of PLC in practice involves other variables that the simulator does not consider. Because this is a specific communication technology, which is significantly affected by, among other things, the environment and the condition of wires, it is necessary to define a number of variables for accurate simulations, which the default solution of the NS-3 tool does not allow, for example age of cables, ambient humidity, and advanced implementation of the possible attenuation in the long-term communication in the power networks of electricity distributors. The main need for simulations is based on the fact that it is a specific technology, whose behavior is given by a specific location and conditions. Equipping the infrastructure with only a minimum number of the necessary devices to verify functionality and reliability is still expensive.

The NS-3 simulator with the G.hn standard extension was used for the mentioned simulations. New models, topology settings and cable types were defined. Based on the analysis of two methods of approach to channel capacity, a more appropriate method of dynamic bit allocation by bit-error rate has been implemented for the mentioned simulations.

This work defined two experimental scenarios for the NS-3 simulations—LV power line and MV power line. The goal was to determine the channel capacity/communication throughput based on the influence of various parameters such as cable type, frequency band, modulation, and PSD. This throughput was the transmit rate at the application layer considering the UDP/IP protocol. In the first series of the BPLC simulations of LV power lines, the correctness of the transmission speed trend with respect to the distance was verified. The second series of simulations considers MV lines with experimental simulation setup using G.hn implementation, where the input parameters are comparable with the IEEE P1901 compliant BPL modems. The designed topology corresponds with the real smart grid topology of the substations. Results comparing simulation and measurement of this topology are demonstrated.

Detected differences may be caused mainly by the variability of the environment and the specific parameters of all elements in the communication path, such as the specified ambient humidity and the age of cables and junctions. The weakness of the simulation is the inaccurate definition of the physical layer, whose parameters could not be determined from the used modems. There are also differences in the setup of the OFDM technique that could not be determined.

Future work will be focused on improving the simulator for advanced topologies in accordance with PLC under real conditions, especially in smart-grid areas for power distribution and management. Different calculation and definition methods were created for the G.hn PLC module in NS-3. The next steps of this work should improve the overall behavior of the simulated PLC network. The features and algorithms, taking into account ambient conditions as humidity, temperature, and cable age, should be considered. It is also desirable to perform advanced and long-term measurements of either real functional or simulated applications that are used in practice. The obtained results could be used for prediction models and integration of these models into the simulator, making it possible to achieve more accurate results for specific locations and topologies with highly variable conditions for PLC communication. The main goal was achieving a correct trend of simulations, corresponding with the theoretical assumptions which allows the simulation of real smart grid systems under real conditions in comparison with measurements.

## Figures and Tables

**Table 1:** Papers dealing with the use of network simulators in general.

Ref.	Year	Content Focus	Content Type
[40]	2020	Comparison of routing protocols under NS-2 and NS-3	Simulations
[43]	2018	Comparison of network simulators	Comparative study
[44]	2020	SLR of main research areas in which NS-3 has been used	Literature review
[45]	2015	Simulations of routing protocols for MANET in NS-3	Simulations
[46]	2017	Simulations of routing protocols for MANET in NS-3	Simulations
[47]	2018	Routing protocols under black hole attack for MANET in NS-3	Simulations

**Table 2:** Papers dealing with simulations with focus on the line type.

Ref.	Year	Content Focus	Simulator	Line
[23]	2013	Simulation and modelling of a PLC channel	NS-3	LV
[50]	2016	Simulation of complex topology	NS-3	LV
[48]	2013	Simulation of simple topology with noise impact	custom	MV
[51]	2011	Simulation and modelling of the HomePlug AV system	NS-2	LV
[49]	2008	Simulations focused on load impedance, line length, branches	custom	LV

**Table 3:** Papers dealing with BPLC simulation with focus on frequency bands.

Ref.	Year	Content Focus	Band	Line
[52]	2018	Hybrid communications architecture for smart grid	1.8–30 MHz	MV
[48]	2013	Mathematical modelling of PLC channel with noise levels	1–100 MHz	MV
[53]	2007	Research of noise characteristics	0.4–2 MHz	MV
[54]	2016	Simulations of BPLC for AMI architecture	1.8–30 MHz	LV
[55]	2018	Basic PLC simulations in NS-3	–	–
[56]	2019	Advanced BPLC G.hn simulations	1.8–30 MHz	
[57]	2018	Simulation of cooperative in-home PLC network	2–30 MHz	–
[58]	2018	Designing an adaptive OFDM modulation method	2–30 MHz	MV

**Table 4:** Papers dealing with comparison of simulations and measurements.

Ref.	Year	Content Focus	Simulator	Line
[51]	2019	Validated model of HomePlug AV PLC in NS-2	2–28 MHz	LV
[59]	2013	Characterization and modelling of real NPLC channels	50–150 kHz	MV
[56]	2019	BPLC simulations of real topology with repeaters in comparison	1.8–30 MHz	MV

**Table 5:** Papers dealing with performed measurements of PLC systems under real conditions.

Ref.	Year	Content Focus	Band	Line
[60]	2013	Measurement of hybrid smart grid architecture with NPLC	1–20 kHz	LV
[61]	2018	Measurement of HomePlug AV2 system with RFC tests	2–86 MHz	LV
[8]	2019	Measurement of BPLC system on the topology without branches with RFC testing	3–30 MHz	MV
[62]	2015	Measurement of hybrid SCADA architecture with NPLC	36–136 kHz	LV
[63]	2015	Measurement of hybrid architecture including 1x NPLC on LV (2–12 MHz) and 2x BPLC on MV (2–7 MHz; 8–18 MHz)	2–12 MHz 2–18 MHz	LV MV
[66]	2019	Measurement of two BPLC systems (proprietary and standardized) on the line topologies without repeaters	3–13 MHz 2–30 MHz	LV/MV LV/MV

**Table 6:** SNR values with targets of BER considering M-QAM modulations.

Number of QAM *M* States	16	64	256	1024	4096
SNR for target BER 10^−3^ dB)	11	15	19	24	29
SNR for target BER 10^−5^ dB)	13	18	22	27	33
SNR for target BER 10^−7^ (dB)	15	20	25	30	35

**Table 7:** Parameters and coefficients for implementation of MV management.

Cable Type	*L* [H/m]	*C* [F/m]	*A*_R_ [Ω/(m · Hz^2^)]	*B*_R_ [Ω/(m · Hz)]	*C*_R_ [Ω/m]
AXEKCY	5.7 × 10^−7^	4.5 × 10^−10^	−4.58 × 10^−15^	1.1 × 10^−8^	8.7 × 10^−3^
AXEKVCEY	5.2 × 10^−7^	3.2 × 10^−10^	−4.58 × 10^−15^	1.1 × 10^−8^	8.7 × 10^−3^

**Table 8:** Input channel parameters of modulation sweep.

Parameter	Value
Access method	CSMA/CA
Cable type (LV)	NAYY 4 × 150 SE
Bandwidth	25 MHz
Modulation	QAM4–QAM4096
Transmit PSD	−55 dBm/Hz
Background noise	−80 dBm/Hz

**Table 9:** Input channel parameters of (**a**) transmit PSD sweep; (**b**) cable cross-section sweep.

(a)	
Parameter	Value
Cable type (LV)	NAYY 4 × 150 SE
Bandwidth	25 MHz
Modulation	QAM1024
Transmit PSD	−55; −65; −60; −70 dBm/Hz
Background noise	−80 dBm/Hz
**(b)**	
**Parameter**	**Value**
Cable cross-section (LV)	NAYY 4 × M SE
M = 50; 95; 150; 185; 240	
Bandwidth	25 MHz
Modulation	QAM1024
Transmit PSD	−55 dBm/Hz

**Table 10:** Input channel parameters of (**a**) cable type sweep; (**b**) bandwidth sweep.

(a)	
Parameter	Value
Cable type (LV)	CYKY 3 × 2.5; NAYY 4 × 150 SE
Bandwidth	25 MHz
Modulation	QAM1024
Transmit PSD	−100 dBm/Hz
Background noise	−80 dBm/Hz
**(b)**	
**Parameter**	**Value**
Cable type (LV)	NAYY 4 × 150 SE
Bandwidth	25; 50; 100 MHz
Modulation	QAM1024
Max. transmit PSD	−100; −85; −55 dBm/Hz
Background noise	−100 dBm/Hz

**Table 11:** Input channel parameters of the experimental simulation of the real BPL topology.

Parameter	Value
Access method	CSMA/CA
Cable type (MV)	AXEKCY; AXEKVCEY
Bandwidth	25 MHz
Modulation	QAM64
Transmit PSD	−55 dBm/Hz
Background noise	−105 dBm/Hz

## Data Availability

Not applicable.
